# Gender equity and emotional labour in the workplace: an ethnographic study

**DOI:** 10.3389/fsoc.2026.1742870

**Published:** 2026-03-11

**Authors:** Gonzague Isirabahenda, Lucian Marina, Bogdan Nicolae Mucea

**Affiliations:** Department of Social Sciences, "1 Decembrie 1918" University of Alba Iulia, Alba Iulia, Romania

**Keywords:** call centers, education–job mismatch, emotional labour, gender inequality, precarity

## Abstract

Romania’s recent economic growth has positioned it as a competitive player in the European Union. However, this macroeconomic progress contrasts with the persistent labour market challenges faced by recent university graduates, particularly in the outsourced services sector. This study investigates the gendereddimensions of education-job mismatches and emotional labour among young graduates employed in Romanian call centres. This study employs 3 years of ethnographic fieldwork (June 2021–June 2024) to investigate how the organisational structure of outsourced customer support creates and reinforces gender inequities, frequently resulting in unstable and limited career trajectories for female employees. Through participatory observation and semi-structured interviews with customer support representatives (CSRs) and their supervisors, this study identifies the often-invisible mechanisms embedded in daily routines, performance metrics, and management practices that perpetuate structural disadvantages within the digital workplace. The findings argue that the work processes in outsourced call centres are characterised by standardisation, emotional labour, deskilling, and rigid hierarchies, which significantly contribute to gender inequality and precarious career paths, especially for young and educated women. Often, the roles of CSRs do not fully utilise employees’ potential, resulting in dissatisfaction, burnout, and high turnover rates. By analysing the intersection of emotional labour and gendered expectations, this study makes a significant contribution to the field of sociology, specifically in the areas of work and employment, offering insights into the structural mechanisms that perpetuate inequality in the contemporary Romanian Business service sector.

## Introduction

The transition towards a service-based economy is a long-standing trend already evident in Romania, where the services sector comprises 51% of total employment (EURES, 2023). Romania has emerged as a key destination for European business service providers. By 2023, Romania ranked 3rd in Europe and 13th globally on AT Kearney’s Global Services Location Index (Kearney, 2023). The top four sectors within the Business Services Sector (BSS) are banking, insurance, and financial services; technology and telecommunications; business and professional services; and industrial and consumer goods (ABSL, 2018). This demonstrates the significant transformation of the Business Services Sector in Romania in recent years.

Many developed countries use outsourcing as a global strategy to enhance their economies, often shifting jobs to countries such as Romania. Outsourcing, a process that involves contracting specific functions of a company to either an employee or another firm, is a global phenomenon that allows tasks to be performed more efficiently and cost-effectively (Peck, 2017). In recent decades, outsourcing has become a priority for many advanced countries. Call centres represent a modern form of outsourcing, relying on a skilled, cost-effective workforce to deliver globally consumed services and information. This trend is primarily due to the various benefits outsourcing offers, which often outweigh potential drawbacks (Government of Romania, 2022).

Globalisation has been a longstanding phenomenon, although it was not always identified as such. It has manifested in various forms in human societies and the job market (Mucea, 2021). This observation is particularly relevant in the Romanian context, where multinational companies (MNCs) have played a significant role in shaping the development of Romania’s higher-education sector, primarily by driving the demand for highly skilled professionals (Tarlea, 2019). Beyond creating employment opportunities in local markets, MNCs also provide their employees with substantial financial and nonfinancial benefits. A major advantage of working for an MNC is the extensive training and professional development opportunities they offer (Dura and Drigă, 2017), which contribute to high standards of skill and overall job satisfaction. There is ample evidence of knowledge spillover associated with MNC employment. Jobs in these companies typically provide competitive salaries and opportunities for knowledge transfer, leading to improved living standards and greater job satisfaction (Tiron Tudor and Ivan, 2021). Additionally, MNCs introduce their organisational culture into the local context. They often promote a more meritocratic environment that rewards individual achievements rather than nepotism. This approach has the potential to enhance upward mobility for employees from disadvantaged socioeconomic backgrounds, particularly women and young workers in societies with a history of communism, such as Romania (Grigore et al., 2023).

The Romanian economy is experiencing growth, having more than doubled in size, moving from the 16th position among EU member states in 2010 to the 12th in 2022. Romania’s GDP growth rate is significantly higher than that of other European countries (Eurofound, 2024). This growth underscores the importance of understanding post-university life, as it is a positive development for recent university graduates and potentially increases labour demand. However, considering relevant data on the labour market, an assessment of the graduate job market in Romania revealed that the career trajectories of young university graduates are more uncertain than ever before. The disparity between graduate supply and skill demand in Romania has intensified, highlighting the gravity of this situation (World Bank, 2023).

Despite numerous narratives on decent work, Romanian literature on the sociology of work and employment has largely overlooked the gendered facets of outsourced work. Romania, a major outsourcing hub, frequently employs recent university graduates in positions misaligned with their educational qualifications. Furthermore, multinational call centres are predominantly staffed by female customer service representatives, whose experiences are shaped by entrenched societal norms and organisational dynamics (Pantea, 2025). The ongoing disparity between educational attainment and employment opportunities for recent university graduates in Romania, combined with organisational practices in multinational call centres, contributes to precarious career trajectories for young educated women and diminishes the value of their higher education (Isirabahenda, 2025).

This study examines how recent university graduates in Romania’s outsourced customer support sector experience gender inequality and emotional labour. It analyses how call centre practices contribute to gender inequity and influence the careers of young, educated women. The study employs ethnographic methods and a gender-focused approach to work and employment. It identifies work processes and systems in outsourced call centres that produce gendered outcomes and uncertain career paths. The article is structured in three parts: the first review gender equity and emotional labour literature, outlines the theoretical framework, methodology, and research design; the second presents the findings; and the third discusses the results and provides conclusions.

## Review of literature

The feminisation of call centre work represents more than a demographic pattern; it is a systemic outcome of gendered labour market sorting and organisational expectations about who is best suited for customer-facing work. These roles rely on intensive interactional and emotional demands, such as empathy, de-escalation, attentiveness, and politeness, qualities routinely coded as feminine and used as the basis for recruitment and role allocation (Hultgren, 2017). Crucially, this feminisation does not translate into the organisational power. Women in frontline positions often remain concentrated in routinised, monitored roles, while pathways to managerial authority are limited (Scholarios and Taylor, 2011). This review examines the mechanisms that reproduce this occupational segregation and connects them to the dynamics of emotional labour and gendered career trajectories, specifically within the context of Romania’s outsourced service sector.

### Gender equity: global trends and the Romanian context

Gender inequality in the workplace remains a critical and widely debated issue in organisational research (Carli and Eagly, 2016; EIGE, 2024). Empirical evidence consistently demonstrates that discriminatory practices constrain women’s career trajectories, limiting their access to opportunities and progression into leadership roles (Johns, 2013). Globally, women held only 33.7% of leadership positions in 2023 (OECD, 2023), a gap perpetuated not only by unequal promotion but also by occupational sorting into roles with limited authority.

Despite decades of progress, women remain less likely to participate in the workforce and often experience lower job quality across OECD (2017) countries and gender inequality persists in the workplace (OECD, 2025). Gender injustice, combined with work-related stressors such as verbal abuse, social exclusion, and psychological intimidation, especially in call centres, contributes to emotional distress, reduced self-esteem, increased psychological strain, job dissatisfaction, and uncertain career paths (Einarsen et al., 2020). Workplace bullying further impairs mental health, stifles innovation, and reduces organisational performance (Jung et al., 2023). Additionally, male AI users, particularly in the financial sector, reported greater benefits from AI adoption than female users. This disparity is partly due to men’s higher representation in management roles, where attitudes toward AI are more favourable (Lane et al., 2023).

The European Working Conditions Telephone Survey (EWCTS, 2021) shows that women and frontline workers are disproportionately exposed to adverse social behaviours at work, leading to higher risks of burnout, exhaustion, anxiety, and depression. OECD data (2023) also indicate that women are much more likely to leave the labour market after becoming mothers, highlighting the ongoing structural barriers to work–life integration. Thirty years after the Beijing Declaration, women and girls continue to face significant gender discrimination, especially when compounded by exclusion related to disability, race, age, income or sexual orientation. Intersectionality further complicates these disparities (United Nations, 2025).

In Romania, this inequality is exacerbated by a post-communist political economy undergoing rapid service sector growth. Despite a formal legal and institutional framework for gender equality shaped by EU membership and international commitments (World Bank, 2019), significant implementation gaps persist (Robayo-Abril et al., 2023). Cultural norms, weak institutional coordination, and inconsistent policy enforcement impede substantial progress. Workplace stress is a significant occupational health concern in Romania that impacts employee wellbeing and organisational success (Eurofound, 2025). Persistent gender equity barriers limit women’s leadership representation and reduce organisational diversity and performance. Addressing gender inequality could increase Romania’s GDP by 8.7% by 2030 (McKinsey and Company, 2021).

Romania’s Gender Equality Index score of 57.5 in 2024, while improved, remains the lowest in the EU and 13.5 points below the average (EIGE, 2024). This score is 13.5 points below the EU average, underscoring persistent structural and cultural barriers to gender parity. Notably, the country scores particularly low in the domains of power (32.8) and violence (36.5). The table below summarises the 2024 indicators of gender equality in Romania.

These scores reveal a societal tension. While Romanian social norms show support for gender equality in influence and authority (low power score), call centres remain structurally hierarchical and male-dominated in leadership. This creates a normative conflict that can heighten women’s sense of injustice. Similarly, the low violence score suggests social disapproval of aggression; however, when female CSRs face verbal abuse, they must manage the added emotional burden within a culture that officially rejects such behaviour. This indicates that structural and organisational practices, not broader social attitudes, are the primary drivers of workplace inequity.

The Romanian labour market exhibits clear gender segregation. Women constitute the majority in service and sales (67.0%), clerical support (65.8%), and professional occupations (59.7%) (National Institute of Statistics, 2025), often in essential, public-facing, or care-oriented roles. This pattern is replicated in the outsourced business services sector, where multinational organisational models intersect with local gender norms, creating a strategic site to assess how economic growth and equality policies translate into work outcomes for young, educated women. Call centres, in particular, offer a strategic setting to assess how gender equality policies and economic growth affect work outcomes.

### The gendered structure of call centre work

Research has identified three interrelated themes that explain the persistent gender segregation in call centres. (1) A dual-track occupational structure where call centres operate on a systemic, dual-track model: a feminised frontline of routinised, emotionally intensive roles and a masculinised managerial tier (Scholarios and Taylor, 2011; Rodriguez and Guenther, 2022). This division often begins pre-hire through gendered self-selection, referral networks, and recruiter bias (Fernandez and Sosa, 2005); (2) Gendered framing of skills, i.e., the concentration of women in frontline roles is driven by an ideology that frames key customer service skills such as empathy, conflict resolution, and politeness as inherently feminine (Ortiz, 2022). This framing renders the intensive emotional labour required both invisible and undervalued, treating it as a natural extension of femininity rather than a professional skill; (3) The risk of technological reinforcement: Emerging research warns that without a critical gender perspective, new technologies such as AI risk automating and reinforcing existing biases rather than disrupting traditional segregation (Ortiz, 2022).

Empirical studies from Australia (Mathies and Burford, 2011), Scotland and Denmark (Hultgren, 2017), India (Jensen, 2012), and the Philippines (Friginal, 2009) suggest that women are both perceived and often perceive themselves as excelling in the interpersonal and linguistic aspects of their jobs. This gendered framing renders the intensive emotional labour required both invisible and undervalued, treating it as a natural extension of femininity rather than as a professional skill. This perception justifies the overrepresentation of women in these roles and the systemic undervaluation of their work.

Gender roles in call centre work differ worldwide. In the Global South, especially in India, the Philippines, and South Africa, call centres can provide women with steady pay, financial independence, and access to night shifts that challenge traditional limits on women’s movement. However, these jobs are not without problems. Women often face moral judgment, safety risks, and strict workplace control (Jensen, 2012; Noronha and D’Cruz, 2017). In Europe and North America, call centre jobs are becoming less secure, with few chances for promotion and more algorithmic management that limits long-term career growth (Brophy, 2017; de Matos, 2024).

New evidence also questions the notion that digital monitoring and scripts eliminate gender bias. Tools such as emotion AI, performance dashboards, and strict scripts often make emotional labour even harder. Women face a double bind: if they stick to the script, they are called cold, but if they personalise their service, they are criticised for being inefficient. When men show empathy, it is more often seen as special rather than expected (Roemmich et al., 2023; Lisdero, 2019).

Most existing research is concentrated in Western or established offshore contexts, limiting our understanding of how these dynamics manifest elsewhere. Romania’s post-communist context is marked by a shift from state socialist female labour promotion to a neoliberal market economy, creating unique gender norms and vulnerabilities (Geambaşu, 2016). Pantea (2025) argues that the sector fosters a depoliticised workforce by emphasising individual responsibility and maintaining unstable job structures, often exploiting young, educated women’s need for employment. The Romanian case serves as a crucial theoretical testing ground, demonstrating how global, standardised call centre models reconstitute systemic gender segregation within a distinct socio-economic landscape. Thus, emotional labour becomes a key mechanism for enacting and experiencing workplace inequality.

### Emotional labour

Emotional labour, the process of managing feelings and expressions to fulfil job requirements (Grandey, 2000), is central to CSR roles. Organisations enforce display rules, encouraging employees to exhibit cheerful, friendly, and patient demeanours to positively impact client interactions (Humphrey et al., 2015).

In CSR roles, their ability to regulate emotions is a powerful tool that empowers them to handle CSR responsibilities effectively (Isirabahenda, 2024). van Jaarsveld and Poster (2013) contended that emotional labour in call canters entails the regulation of emotions via verbal cues during customer interactions, and factors such as work conditions, organizational environment, customer behaviour, and individual characteristics such as personality, gender, and race can significantly impact emotional work performance. The use of technology and the global nature of call centre operations continue to shape the dynamics of emotional labour in this context.

The consequences of this are profound. CSRs must manage customer complaints and verbal aggression while meeting strict performance targets, often leading to emotional dissonance, a disconnect between the felt and displayed emotions (Grandey and Gabriel, 2015). This dissonance, coupled with high stress, electronic surveillance, and low autonomy, contributes to emotional exhaustion, burnout, and high turnover (Chen et al., 2019; Rod and Ashill, 2013; Woydack, 2019).

Emotional labour is often seen as a way for women to gain recognition or get promoted, but recent research shows that it usually keeps them in certain roles. Women are often placed in customer service jobs because empathy and emotional control are seen as natural traits for women rather than skills. This creates a “glass cage,” where being good at emotional work blocks access to technical or strategic jobs, which are still seen as masculine. Even when women move up, they are more likely to end up in “soft” management roles, such as coaching or wellbeing support (Akdemir, 2023; de Matos, 2024).

Research on emotional labour highlights both the psychosocial costs and organisational benefits of managing emotions in customer service roles. This also prompts a broader question: How do gender and organisational power shape these emotional demands? The next section examines the theoretical perspectives on how emotional labour is linked to gendered organisational structures, precarity, and the reproduction of inequality in outsourced service settings.

### Gender inequities and emotional labour: a theoretical lens

To analyse this complex phenomenon, this study integrates several theoretical perspectives. No single theory can fully encapsulate all dimensions of this phenomenon. Sociological approaches to gender equality at work highlight a mix of structural, cultural, and interactional factors. Gender inequities in organisational contexts manifest through wage disparities, leadership gaps, and occupational segregation, creating systemic disadvantages for women. These inequities intersect with precarity theory (Standing, 2011), which conceptualises insecure and low-quality work as a defining feature of contemporary labour markets, disproportionately affecting women through temporary contracts and limited career progression. Symbolic interactionism helps interpret how gender is constructed in everyday CSR interactions, reinforcing stereotypes that women are naturally nurturing and men are leaders (Mastracci and Arreola, 2016). These micro-level interactions shape hiring, promotion, and workplace culture, often resulting in gendered stereotypes and unequal outcomes (Tang and Xu, 2023). Emotional labour theory illuminates the gendered dynamics of emotional labour, recognition, and workplace inequality in customer service roles (Zapf et al., 2014).

Gendered organisation theory (GOT) explains structural and procedural issues. The three key processes from Joan Acker’s GOT (2012) are the gendered division of labour, interactional processes, and organisational logic. The gendered division of labour suggests that organisations assign roles based on gender assumptions (e.g., women in emotional frontline roles and men in managerial positions). Several call centre studies provide clear evidence for this division. For example, some studies report that women are often assigned roles involving emotional labour and caregiving (Woodcock, 2017), supporting Acker’s view that stereotypes about women’s emotional skills influence job assignments.

Interactional processes explore how daily interactions reinforce the gendered power dynamics. This study analyses and illustrates these dynamics in a call centre environment. For example, female CSRs are expected to remain calm and polite even when facing verbal abuse, and supervisors rarely intervene, reinforcing the expectation that women should endure such mistreatment. In terms of recognition, studies show that high-performing women are sometimes labelled as “arrogant,” while male competence is accepted and celebrated. This demonstrates how gendered interactions shape perceptions and rewards, often penalising women for traits valued in men, such as assertiveness (Rowe et al., 2013; van Jaarsveld and Poster, 2013).

The organisational logic process involves underlying, often male-centric, assumptions that shape an organisation’s structure and operations. This study analyses how work organisation reflects these assumptions. The use of scripts and monitoring software standardises tasks and deskills the workforce, which, according to Acker’s theory, helps reproduce gender hierarchies. Women are often stereotyped as compliant workers suited to high-pressure and low autonomy roles (Jenkins et al., 2010). Acker argues that this logic disproportionately disadvantages women, who often have greater caregiving duties. While other processes, such as the gendered construction of symbols and individual identity, are also relevant, the three processes discussed above are most central to the study’s arguments about structural inequality, daily interactions, and work design in the call centre.

To empirically investigate how these theoretical mechanisms, such as gendered division of labour, interactional norms, and organisational logic, manifest and intertwine within the specific socioeconomic context of Romania, this study employs an ethnographic methodology, which is detailed in the following section. Combining the GOT, precarity theory, symbolic interactionism, and emotional labour theory allows for a thorough analysis of how societal norms, organisational policies, and interpersonal dynamics coalesce to produce and perpetuate inequality. Therefore, an ethnographic approach was selected to move beyond surface-level observations and provide a detailed interpretative analysis of the subtle everyday realities of gender and work in Romanian outsourced call centres.

## Methodology

This ethnographic study investigates gender inequality and emotional labour among recent university graduates in Romania, with a specific focus on customer support roles within outsourced call centres. This study examines how organisational work processes contribute to gender inequity in CSR positions.

**Figure 1:**
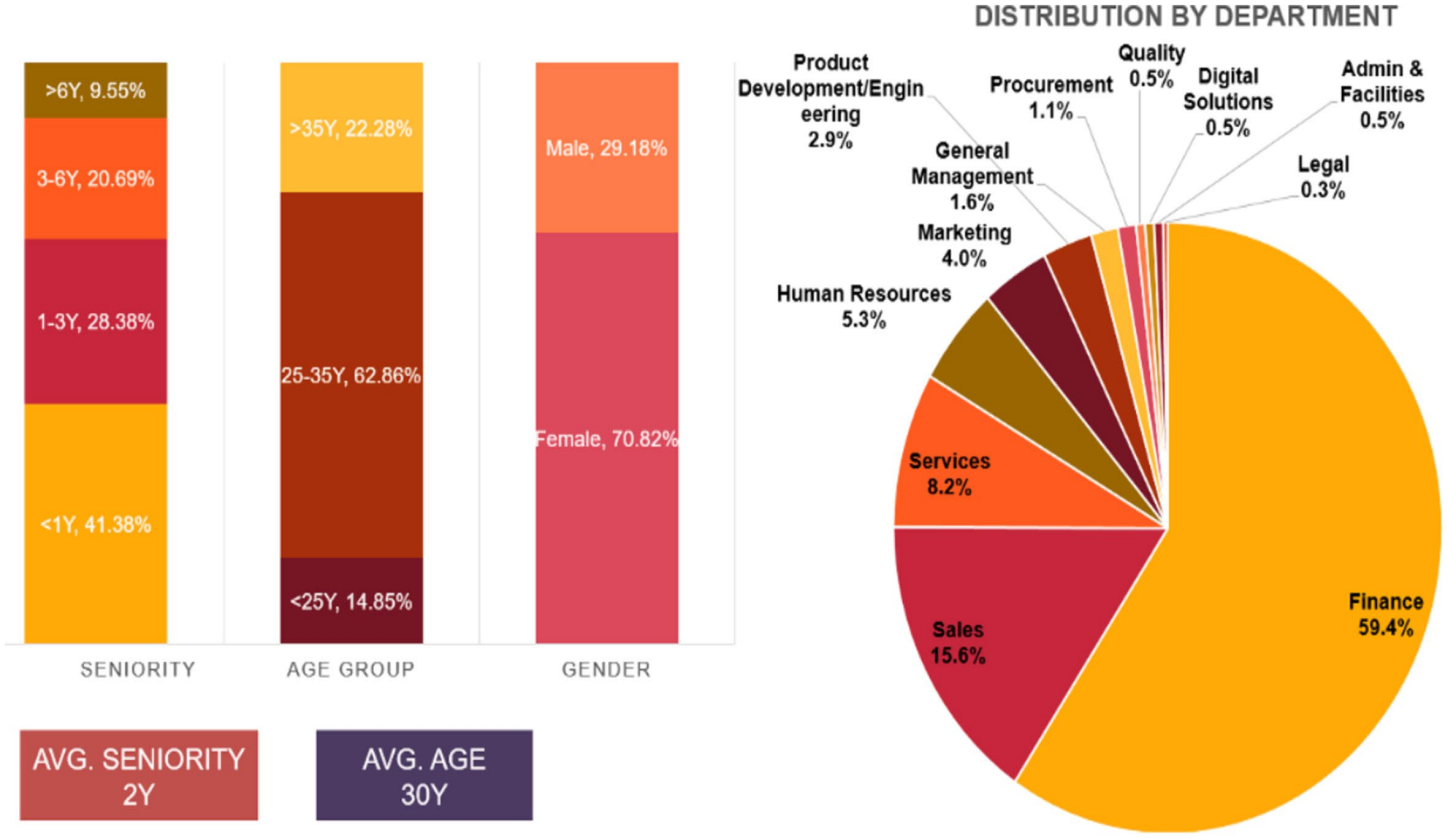
Global corp. employee distribution. Source: Fieldnote, 2022–2024.

Overall, Global-Corp has nearly 270 employees working in different departments: Finance, Sales, Marketing, human resources, services, product development, engineering, IT, digital solutions, procurement, legal, and general management. The finance department was the largest crew member, accounting for approximately 60% of all Global-Corp employees. This is followed by the sales and service departments, which account for approximately 24% of employees. In terms of gender distribution, as shown in the Figure 1, females represent 71% of the Global-Corp employees.

To capture both individual experiences and organisational practices, a multi-method qualitative approach was employed, data were collected between June 2021 and June 2024 combining in-depth interviews with participant observation. Semi-structured and unstructured interviews were used to explore personal narratives, perspectives, and meanings attached to gender roles and emotional labour. These direct verbal interactions were designed to elicit detailed accounts of participants’ experiences, perceptions, and emotions. All interviews were audio-recorded and transcribed to preserve the authenticity of the participants’ accounts. The researcher facilitated these conversations by guiding the topics while allowing participants to express themselves freely.

Complementing the interviews, participant observation was conducted to examine workplace culture, organisational practices, and gendered behaviours *in situ*. This method provided real-time observational evidence of routines, interactions, and norms that might not have been revealed in interviews alone. By actively engaging in the call centre environment, the researcher gained an insider perspective, reduced the observer effect, and captured non-verbal cues and unspoken dynamics through detailed field notes. The concurrent use of both methods enabled triangulation, strengthening the study’s validity by comparing spoken accounts with observed behaviours and yielding a richer and more nuanced understanding of the research context.

### Data collection and participants

Participants were purposively selected to illuminate the issues of gender inequality and emotional labour in outsourced customer support. Interviews were scheduled at the participants’ convenience, predominantly outside working hours or on weekends, and lasted for 40–60 min. In total, 61 semi-structured face-to-face interviews were conducted: 50 with CSRs, seven with team leaders, and four with managers. Additionally, informal conversations were held with approximately 60 entry-level employees during fieldwork. These exchanges informed contextual interpretation but were not analysed as primary data. The participant demographics are summarised in the Table 1.

**Table 1:** Indicators of gender equality in Romania.

	Work	Money	Knowledge	Time	Power	Health	Violence	Index
RO	67.5	72.8	55.4	69.2	32.8	70.4	36.5	57.5
EU	74.2	83.4	64.2	68.5	61.4	88.6	31.9	71.0

Source: EIGE (2024).

The final sample comprised 50 CSRs (36 women, 14 men), seven Team Leaders (five men, two women), and four managers (three men, one woman). Participants varied in gender, age, education, and career stage, providing a comprehensive perspective on how organisational practices and emotional labour shape the experiences of young, educated women in CSR roles and influence gendered career progression. Of the participants, 35 were aged 18–25 years, and 26 were aged 26–42 years. The participants’ education levels were nearly evenly split, with 33 holding bachelor’s degrees and 28 holding master’s degrees. Most CSRs (33) and six of the nine team leaders and managers held degrees in Humanities and Social Sciences; the remaining three had professional or applied degrees.

### Data analysis

Qualitative data, comprising interview transcripts and extensive field notes, were transcribed, resulting in more than 200 pages of text. This corpus was imported into NVivo to facilitate structured coding and analysis. Each interview was transcribed verbatim and subjected to multiple rounds of iterative reading, with analytical notes taken throughout to ensure a comprehensive and nuanced understanding of the data. Data collection and preliminary analyses were conducted concurrently to maintain the representativeness and analytical flexibility (Taylor et al., 2016).

This study employed a reflexive thematic analysis approach, as guided by Braun and Clarke (2022). This method acknowledges the researcher’s active role in knowledge production, allowing themes to emerge analytically from coded data rather than being imposed by pre-existing categories. Its flexibility made it particularly suitable for the research questions, while its structured six-phase process ensured rigour. The analysis was as follows: We began by immersing ourselves in the data (Step 1) through transcription, repeated reading of the field notes and transcripts, and writing down initial ideas. Next, we systematically generated initial codes (Step 2) by tagging the salient features across the entire dataset. These codes were then collated into potential themes (Step 3), which were refined and reviewed in a recursive process (Step 4) to ensure that they formed a coherent pattern relative to the coded extracts and the entire data set. Subsequently, we defined and named the themes (Step 5), articulating their essence and scope. Finally, we produced a report (Step 6), weaving the analysis into a scholarly narrative that illustrates the significance and prevalence of each theme with compelling data excerpts, thereby providing a rich and credible account of our findings.

### Ethical considerations and trustworthiness

In line with Creswell (2013), this study adhered to fundamental ethical principles, ensuring the participants’ rights to anonymity, informed consent, and voluntary participation with the option to withdraw at any time (Flick, 2022). To ensure the study’s validity and trustworthiness, four key criteria were addressed (Polit and Beck, 2010): (a) Credibility was bolstered through prolonged engagement (36 months of on-site observation), thick description, and comparison with findings from similar studies (Denzin and Lincoln, 2011); (b) Dependability was achieved via rigorous methodological documentation, pilot interviews, the researcher’s expertise (Liamputtong, 2019), and debriefing sessions to minimise bias; (c) Confirmability was ensured through the use of reflexive journals and extensive triangulation across multiple data sources and methods; (d) Transferability was enhanced by providing a rich, contextual analysis of Romania’s business service sector, allowing readers to assess the applicability of findings to other settings (Maxwell, 2021). This multilayered approach ensured that a transparent qualitative research process was followed.

Theoretical saturation was achieved through extended fieldwork, diverse participant selection, ongoing and iterative data analysis, and constant comparison, with data collection continuing until a comprehensive understanding of gender inequality and emotional labour in this context was reached. Applying this methodological approach, the analysis identified three central themes that structure the following findings and discussion: (1) the gendered distribution of roles and management positions, (2) the gendered expectations placed on employees within CSR functions, and (3) the specific work processes and organisational mechanisms that institutionalise gender inequities.

## Findings

The study’s findings highlight some aspects of gender inequality in the context of CSR’s role and career progression. The data analysis identified three key themes: (1) gendered distribution in CSR roles and management, (2) gendered workplace expectations within CSR roles, and (3) work processes and organisational mechanisms that contribute to gender inequities.

### Gendered distribution in CSR roles and management

The analysis of data uncovered distinct gender patterns in CSRs and management roles. In reference to Table 2, of the 61 participants, 39 were women and 22 were men, indicating that women comprised approximately 64% of the total participants and men comprised 36%. In the context of Entry-Level CSR positions, among the 50 CSRs involved in this study, 36 (72%) were women and 14 (28%) were men. This underscores the gendered concentration of women in entry-level roles, aligning with broader trends in customer service and care-related employment. In examining the gender distribution within managerial roles, 11 managers and team leaders were identified: four managers (three men and one woman) and seven team leaders (five men and two women). Men occupy eight of the 11 managerial positions (73%), whereas women occupy only three (27%). This indicates a gender imbalance in leadership, with men being disproportionately represented in higher-status roles, despite women constituting most of the call centre workforce. In summary, women predominantly occupy lower-tier roles (CSRs), whereas men dominate managerial positions. This distribution aligns with gendered organisation theories (Acker, 2012), where workplace structures perpetuate gender hierarchies through role assignments and promotional practices.

**Table 2:** Demographic data of participants.

Participants career stage	Gender		Age categories		Ed. level		Field studied		
	F	M	18–25	26–42	BA	MA	Professional and applied sciences	Humanities and social sciences	Formal sciences
CSRs	36	14	32	18	37	13	17	27	6
TLs	2	5	2	5	4	3	2	4	1
Managers	1	3	1	3	2	2	1	2	1
Total	39	22	35	26	33	28	20	33	8

Source: Primary data, June 2021–June 2024.

In addition to the high proportion of women in CSR roles but low representation in supervisory or managerial roles, this study’s findings noted the difficulties faced by CSRs daily, which were heightened by considerable stress due to ambiguous duties and expectations, leading to confusion about responsibilities, clients, and company operations. There was a discrepancy between the advertised job descriptions and the actual responsibilities of the CSRs. For instance, the job description emphasised adaptability and flexibility and promised a flexible work schedule for employees. However, the actual role requires inflexible work, a high emotional labour load, and dispute resolution. This observation was confirmed in other BPO cases, in which CSRs were required to possess a significant selling ability in addition to their previously described job duties.

### Gendered workplace expectations in the CSR roles

This study explores the gendered dimensions of the transition from higher education to employment, drawing on narrative accounts from Romanian CSRs. The findings reveal both explicit and implicit gendered expectations shaped by societal norms, cultural assumptions, and workplace dynamics.

#### Gendered roles in family and independence

The narratives suggest that women are often perceived as requiring prolonged dependence, particularly in early adulthood. For instance, Oana’s account illustrates the societal expectation that women may be less prepared for independent living:

*“The University made me more responsible because I came here alone; I left home at the age of 18 to study here. However, I had to manage on my own and go to classes, as there was no one left to pull me or say, Oana, do that, take care of that, do not forget this.”* Oana, 18–25 years old; BA

In contrast, male participants such as Andrei and Grigore described their university experience as a catalyst for rapid maturation and autonomy:

*“Before attending university, I found it difficult to understand the world. However, the university provided me with a broader perspective, and as a result, I have evolved into a mature man capable of living independently and supporting myself.”* Andrei, 18–25 years old; BA

*“After obtaining my bachelor’s degree, I moved to Spain and found that I had to adjust to and become self-sufficient in a new environment. Over the course of one-and-a-half years, I was pleased to realise that I had matured into a responsible adult.”* Grigore, 26-42 years old; MA

Andrei and Grigore described their university experience as a path to becoming “mature men capable of living independently.” These accounts reinforce the normative expectation that men should swiftly transition to self-sufficiency and adult responsibilities.

#### Career aspirations and gendered job imagery

Female participants often envisioned alternative career paths that aligned with traditional feminine roles. Monica, for example, considered becoming a stewardess, a profession historically associated with appearance and service:

*“After a three-month course, I will be equipped with everything I need to know about the profession. The course costs roughly 1500 euros, and students are guaranteed to receive a job at the end.”* Monica, 18–25 years old; BA

Conversely, male participants, such as Mihai, referenced technical or manual labour and emphasised their confidence and responsibility in that role, reflecting gendered occupational norms:

*“I would have continued working as an auto mechanic in France. I used to work there during vacations or whenever I had the free time. My father has a vehicle repair business in France, and I have been working there since I was 16.”* Mihai, 18–25 years old; BA

#### Gendered emotional labour

Our ethnographic data reveal a pronounced gender disparity in emotional labour expectations within CSR roles. Female CSRs are expected to demonstrate greater emotional resilience, adaptability, and self-sacrifice than their male counterparts. This is evident in Iulia’s narrative, which exemplifies the internalisation of emotional labour norms:

*“Recognising the necessity of fulfilling both customer and employer requirements often demands self-sacrifice. I frequently allocate additional time to complete tasks and shift my break times to accommodate such situations. Simultaneously, I continuously engage in personal and professional development to prepare myself for the challenges of work-related pressure and enhance my interpersonal abilities.”* Iulia, 26–42 years old; BA

Iulia’s account reflects how women are expected to absorb emotional strain, adjust their schedules, and invest in self-improvement to meet workplace demands. Her experience illustrates the gendered burden of emotional labour, where dedication is often taken for granted rather than rewarded. In contrast, male CSRs tend to frame their experiences around skill acquisition and strategic growth, emphasising performance over emotional adaptation. Samuel’s narrative reflects this orientation:

*“The more complex the situations I faced and handled, the more my skills were enhanced… We help new employees to enhance their customer support skills.”* Samuel, 18–25 years old; BA

Samuel’s progression from novice to mentor is framed as a technical achievement, reinforcing the perception of men as rational and capable leaders. Samuel’s quote reflects a performance-oriented mindset, where emotional labour is reframed as an opportunity for skill enhancement and leadership. His contributions are seen as strategic, while women’s efforts, such as Iulia’s, are often interpreted through the lens of self-sacrifice rather than competence.

This study reveals the gendered recognition and misinterpretation of female excellence. Recognition within the CSR environment also appears to be gendered. While male competence is generally accepted and celebrated, high-performing women are sometimes perceived as arrogant or overreaching. Despite consistently meeting individual targets and outperforming her peers, Iulia was viewed as arrogant rather than exemplary by some colleagues. This reflects a double bind in which women must excel while remaining modest to avoid social penalties.

This study highlights that support and mentoring roles are gendered in their representations. Women are often positioned as nurturers, expected to provide emotional and interpersonal support, whereas men are portrayed as strategic thinkers and technical experts. Samuel’s statement reinforces this dichotomy:

*“I became familiar with controlling calls and managing my portfolio as needed.”* Samuel, 18–25 years old; BA

Similarly, Lucian’s narrative reflected a future-oriented and rational approach to career development:

*“After completing my undergraduate degree, I found myself at a crossroads, unsure whether to continue my education or seek employment in the field. Simultaneously, I lacked clarity on the specific job I desired and wanted to engage in the job market for. Now, I am a different person and can figure out what skills will be needed in five years.”* Lucian, 18–25 years old; BA

These male narratives emphasise strategic thinking and autonomy, contrasting with the emotional adaptability expected of female CSRs.

This study reveals the adaptability and emotional resilience of female CSRs. Female CSRs are expected to emotionally adapt to workplace culture, often without adequate support or preparation. Monica’s account illustrates this:

*“The transition from university to a job was challenging. Navigating job searches and adapting to workplace culture require the development of new skills and adjustment to new expectations. Workplace behaviours and expectations are distinct from those observed in university settings; however, I adapted and thrived in this new environment.”* Monica, 18–25 years old; BA

Monica’s experience highlights the emotional labour of adaptation, where women must quickly learn and conform to workplace norms, often under pressure. In contrast, male CSRs are expected to overcome uncertainty through future-oriented planning rather than through emotional adjustment.

#### Social mobility and gendered narratives

Women’s mobility is frequently framed within familial and relational contexts. Ana’s experience with housing and financial independence is mediated by her parents’ reactions:

*“During my study period at Babes-Bolyai University, I witnessed the difficulties associated with renting accommodation. I had no idea how to own an apartment in a large city such as Cluj-Napoca. Over time, I can say that I have matured enough to take out mortgage loans. I received aid from my parents, who were speechless when I mentioned the idea, and I am about to pay off my mortgage and no longer have to worry about the rent.”* Ana, 27 years old; MA

Ana’s story about renting and taking out a mortgage is framed through her relationship with her parents and their surprise, suggesting that financial independence for women is still seen as exceptional in the culture. However, men’s mobility is portrayed as being driven by personal ambition and global engagement. For instance, Tom shares social benefits through his journey to develop friendships, community engagement, and global connections when asked about the benefits he considers the university has so far:

*“I had no idea that, at a certain point in my life, I would develop friendships and the nature of the community in which I would engage before entering university. I had a dropout mindset because no one believed that I would stick to my studies and graduate from university. Ultimately, because of multicultural events and projects, I have friends from all over the world. In other words, I have become a global citizen*. *”* Tom, 26 years old; MA.

Vlad, one of the CSRs, discussed how his university experience familiarised him with Romanian politics and sparked his interest in social injustice.

*“My university experience significantly influenced my perspective on politics in Romania and worldwide. I have compelling arguments regarding globalisation, corruption, poor governance, social injustice, and public health. My local community trusts and admires me for my intelligence and knowledge. I have convinced numerous community members to be vaccinated against COVID-19 and organised events and volunteer projects for teenagers.”* Vlad, 18–25 years old; BA

Tom and Vlad describe their university experiences as gateways to global citizenship and political engagement, reinforcing the idea that men are expected to be outward-facing and socially influential.

### Work processes and organisational mechanisms shaping gender inequities

#### Recruitment and entry mechanisms

Recruitment into CSR roles at GLOBAL-CORP follows a multichannel strategy with a strong emphasis on internal referrals. Among CSRs surveyed, 75% were hired through employee recommendations, 18% via job advertisements on platforms such as Best Jobs and E-jobs, and 7% through direct interactions at career fairs. This distribution highlights the dominance of social capital in the recruitment process. A senior HR executive explained the rationale behind this preference:

*“Our employees are excellent ambassadors for the company; therefore, we highly encourage the use of this method of referral. In addition, we post job offers on various platforms, such as our website, the company's Facebook page, and LinkedIn. We also used job search websites, such as Best Jobs and E-Jobs. Additionally, we actively participate in job-career fairs to promote our programs.”* Elisa, 26–42 years old, HR

Referrals are incentivised using a structured bonus system. When an employee successfully refers a candidate who remains with the company for at least 6 months, they receive a referral bonus ranging from €500 to €1,500, depending on the seniority of the role. Bonuses are disbursed only after the probation period and are subject to statutory deductions.

*“The bonus is paid only after the suggested candidate completes the probation period and is subject to local statutory deductions and regulations.”* HR Specialist, field notes

This system not only reduces recruitment costs but also strengthens internal networks. As one CSR shared:

*“So far, I have recommended three individuals: two former colleagues and my cousin. I am inclined to assist my university peers in identifying new job opportunities; naturally, I receive compensation for my efforts in doing so. Given the various financial circumstances under which my relatives are unable to assist, I frequently find myself in situations that require quick thinking, and I continue to encourage them in the hope of a prolonged stay.”* Ilie, 18–25 years old; BA

While referrals are cost-effective and foster retention, they also create closed recruitment networks that privilege people with strong social ties. This mechanism can inadvertently exclude women and marginalised groups who lack access to male-dominated professional circles, reinforcing systemic gender inequities (Bourdieu, 1986; Rubineau and Fernandez, 2015). Moreover, contractual segmentation exacerbates inequalities. Graduates with master’s degrees are often offered fixed-term contracts, whereas those with bachelor’s degrees are offered permanent roles. This disproportionately affects women, who are overrepresented in call centre roles (Pantea, 2025).

Despite the routine nature of CSR tasks, employers frequently require university degrees, contributing to credential inflation and education-job mismatch. This undermines career progression, particularly among women.

*“Companies rarely offer these positions to non-graduates. CSR functions are designed for individuals with university degrees or at least a BA. If you check the Classification of Occupations in Romania (COR), you will see that this job requires university-level qualifications.”* Vlad, 26–42 years old; BA, TL

However, internal perspectives challenge this requirement:

*“There are few examples of employees without university degrees. Entry-level positions typically do not require a university degree. I have employees with high school diplomas who are doing remarkable work and are eligible for promotions. Some employees hold multiple university and master’s degrees, but their performance is poor.”* Emilia, 26–42 years old, Senior HR

*“I know that some of my colleagues here are university dropouts, others have high school degrees, and I am sure they do not have documents to prove they are university graduates.”* Anet, 18-25 years old; BA

*“You do not need a university degree for this CSR role, but having one is a big plus. No university degree is necessary because we use various accounts receivable software and perform repetitive tasks.”* Andrei, 18-25 years old; BA

These insights reveal a disconnect between formal job requirements and actual performance, suggesting that soft skills, adaptability, and emotional resilience may be more critical than academic credentials for CSR positions.

#### Induction and training practices

The onboarding process at GLOBAL-CORP places significant emphasis on shadowing and informal mentoring, primarily by senior CSRs. This form of work-based learning allows new employees to observe and emulate experienced colleagues and gain practical insights into job organisation, task execution, and workplace dynamics.

*“When employees shadow, they can learn how the job is organised, how concepts are applied, how they can work, what to do, know where to seek help, ask questions, and learn more than what they gain from training alone.”* Ciprian, 18–25 years old; BA

However, this shadowing is unpaid and unrecognised, reflecting broader patterns of gendered care work, wherein women disproportionately perform relational and developmental labour without compensation or formal acknowledgement (Guy and Azhar, 2018). As one senior CSR noted:

*“… I like to teach people, regardless of the time spent doing that, the company does not pay for that.”* Alina, 26–42 years old, field notes

Alina’s experience underscores the invisible labour embedded in CSR onboarding, where mentoring is expected but remains unrewarded. While she expressed a willingness to support new hires, she also highlighted the lack of institutional recognition for her efforts, an issue that mirrors the gendered expectations of emotional and nurturing labour in the workplace. This mirrors the findings of Guy and Azhar (2018), who argue that such unpaid developmental tasks are frequently feminised and undervalued.

Training practices also reveal gender disparities in terms of visibility and advancement. Informal mentoring tends to favour those with stronger workplace networks, which are often male dominated. Female CSRs may have limited access to high-visibility tasks or strategic portfolios, which reduces their chances of gaining career-enhancing experience. For example, although Alina actively mentored new hires, her contributions were not formally acknowledged. In contrast, male CSRs, such as Alex, used shadowing periods to disengage from work:

*“I sometimes breach official working procedures and use the shadowing period to relax.”* Alex, 18–25 years old; BA

This contrast highlights how gendered expectations shape attitudes toward training: women are expected to nurture and support, whereas men may opt out without consequence.

The average induction period ranges from 2 weeks to 1 month, combining brief formal training with observations. However, many CSRs reported that this timeframe was insufficient, leading to stress, confusion, and emotional fatigue.

*“The induction period was brief… new CSRs complained about inadequate training and insufficient time allocated, leading to boredom and stress.”* Ovidiu, 18–25 years old, Junior CSR, field notes

*“When I started working, I experienced considerable stress because my lack of clarity about my responsibilities and expectations left me uncertain and confused.”—* Sofia, 18-25 years old; BA

These challenges disproportionately affect female CSRs, who are expected to excel in emotional labour roles while navigating unclear expectations. As Grandey and Gabriel (2015) note, women in service roles often face higher emotional demands but receive less support and recognition.

The lack of structured and inclusive training programs contributes to gendered skill gaps and limited upward mobility. Without formal recognition or compensation, female CSRs performing mentoring roles may experience burnout and stagnation, while their male colleagues benefit from informal privileges. In summary, GLOBAL-CORP’s induction practices, while effective in some respects, are marked by gender bias in labour expectations, access to mentorship, and recognition, thereby reinforcing structural inequalities in the workplace.

#### Work organisation and processes

Customer Service Representative (CSR) roles at GLOBAL-CORP are defined by standardisation, routinisation, and technological control, resulting in deskilling and diminished autonomy. Deskilling is the process by which highly educated or skilled workers are assigned tasks that underutilize their capabilities, leading to frustration and diminished job satisfaction. Tasks are heavily scripted and mediated through software systems such as GetPaid and Billtrust, which automate the workflows and monitor the performance.

*“We have transcripts to read to ensure that we follow the collection rules. All outgoing calls are standardised… avoid your strategy or have a new idea. I am telling you that all tasks are simplified to the level that anyone can easily handle CSR duties. Various software applications notify managers and team leaders of incomplete or neglected priority tasks.”* Mona, 18-25 years old; BA

Mona’s account illustrates how technological scripting reduces the need for creativity, transforming CSRs into task executors rather than problem-solvers. This is particularly demotivating for university-educated CSRs, whose skills and qualifications are underutilised. This quote highlights how CSR tasks are reduced to scripted routines, leaving no room for innovation or personal judgment. In addition, the skills required to get and do the job differ, and this job role does not require a university qualification; still, almost all CSRs have one or two university degrees. This observation reflects the mismatch between educational attainment and job requirements, which are key indicators of deskilling.

*“My job usually entails continuous calls with clients… I must pay close attention to what my customers say while rapidly turning pages on a screen, adding notes to the system to ensure that all details are accurate and recorded and that the right things have been said in a way that follows the collection policy.”* Sofia, 18-25 years old; BA

Sofia’s quote highlights the cognitive overload and emotional precision required in CSR roles, despite their low status and the repetitive nature of the work. The monotony of daily tasks, such as clearing queues and updating ERP notes, contributes to burnout and disengagement.

*“Our job duties are less specific, and we do not have any control. Neither our team leads nor our managers can resolve the issue of task allocation. I am confronted with various issues that hinder a smooth working environment.”* Corina, 26–42 years old; MA

The findings reveal that CSRs face issues related to portfolio complexity and management pressure. Workload distribution is uneven, with some CSRs assigned high-conflict portfolios involving unresolved disputes and delinquent clients. This reflects how limited decision-making power and rigid task structures contribute to deskilling and workplace dissatisfaction. Team leaders acknowledge the emotional toll but frame it humorously:

*“Today, Alina and Mihai are ready to become either hated people or collectors… May the force be with you!”* Rodica, 26–42 years old; TL

Rodica’s comment underscores the emotional strain and reputational risks associated with certain portfolio types. These assignments often lack transparency and negotiation, reinforcing Taylorist principles of control and efficiency over employee wellbeing. There is also misalignment between skills and tasks

*“Despite having good computers, offices, and recreational facilities, I feel stressed because of excessive work. Consequently, I lost hope for this job and its benefits.”* Iustina, 18–25 years old; BA

Iustina’s quote underscores how infrastructure and the environment do not compensate for the lack of meaningful, skill-aligned employment. The results show the gendered dimensions of control and the performance. The rigid structure of CSR work aligns with Acker’s (1990) Gendered Organisation Theory, which argues that bureaucratic systems reproduce gender hierarchies through routinisation and surveillance. Performance metrics, such as the mandatory 30 + outbound calls per day, create high-pressure environments where women, stereotyped as compliant and emotionally resilient, are expected to meet unrealistic targets.

The findings reveal that CSRs face issues related to emotional labour and identity strain. CSRs are required to display empathy and suppress negative emotions, even in the face of verbal abuse. This reflects Hochschild’s (1983) Emotional Labour Theory, where affective performance is commodified but remains uncompensated and undervalued. Pretending to feel good while in pain is a direct expression of emotional dissonance:

*“Dealing with angry customers can be frustrating. I dislike pretending to feel good when I am in pain. I have to take and receive other calls, and it is mandatory to cover my previous challenging experiences.”* Lucian, 18–25 years old; BA

Lucian and many other CSRs were always required to suppress their personal emotions to maintain a professional demeanour. Lucian’s quote reflects the core of emotional labour: masking genuine feelings to maintain a calm and professional demeanour even when emotionally distressed. In addition, the findings show how CSRs are forced to remain calm despite verbal abuse. This institutional expectation to suppress emotional responses, even in the face of hostility, reinforces the burden of emotional labour. For instance, CSR agents endure verbal abuse without retaliation.

*“Every day, we contact customers for unpaid bills… they sometimes resort to using offensive language, such as f-words and stupid. Worse, our team leaders always advise us to remain calm, avoid arguing with customers, and never hang up on them.”* Ion, 26–42 years old; MA

This quote exemplifies emotional suppression under duress, where CSRs are expected to absorb abuse without reacting, reinforcing the feminisation of affective labour.

Supervisors are aware of these abuses but rarely intervene, reinforcing the feminisation of affective work (Zapf et al., 2021). The expectation to remain calm and composed under duress disproportionately affects female CSRs, who are socialised into emotional regulation roles.

The findings highlight the cultural adaptation and identity suppression practices. For instance, to meet customer expectations, CSRs often abbreviate their names or adopt American accents, practices that impose identity strain and reinforce the cultural hierarchies. These adaptations are more commonly expected from female CSRs, who are perceived as being more flexible and accommodating (Humphrey et al., 2015).

*“Many colleagues abbreviated their names to sound American and tried to imitate American accents to make things easier.”* Erica, 18–25 years old; BA

Erica’s experience shows how emotional labor extends to identity management, requiring CSRs to modify their speech and presentation to meet customer expectations. This reflects cultural emotional dissonance, where CSRs alter their identity and speech to meet customer expectations, often at the cost of authenticity. In addition, our findings illustrate identity strain, a form of emotional labour in which CSRs feel compelled to alter or suppress aspects of their identity to meet workplace or customer expectation. For example, owing to outsourcing, CSRs navigated customer mistrust:

*“Some customers always doubt paying with credit cards when I tell them that our office is in Romania.”* Nadia, 18–25 years old; BA

Nadia’s experience shows how geographic and cultural identity can become a source of tension, requiring CSRs to manage scepticism and maintain professionalism despite being perceived as less trustworthy. Similarly, CSRs endure the emotional burden of misaligned expectations:

*“Customers requested refunds and waivers to lien… they are upset thinking we are slow in resolving their requests and always want to escalate and talk to management.”* Iustina, 18–25 years old; BA

Iustina’s quote highlights how CSRs must navigate blame and frustration directed at them for issues beyond their control, often requiring them to suppress their reactions and maintain a composed identity despite feeling frustrated.

The findings suggest that CSRs must manage customer frustration and mistrust while maintaining composure despite lacking control over the underlying issues. Handling customer mistrust and misunderstanding leads to emotional strain from customer mistrust:

*“Customers wish to avoid paying bill taxes at a specific rate… These issues, beyond our work control, affect our mental and emotional wellbeing.”* Paul, 18–25 years old; BA

These examples highlight the emotional toll of being held accountable for systemic issues, requiring CSRs to remain composed while navigating customer frustration and mistrust. This study reveals how CSRs face emotional dissonance and burnout in daily work practices, and the manifestation of emotional work was realistically noticeable as many CSRs perceived unfair treatment and disrespect from customers, team leaders, and direct supervisors.

*“Suppressing certain emotions to satisfy clients is unhealthy and is likely one of the major causes of CSR downturns.”* Felip, 26–42 years old, Senior CSR, field notes

These reflections align with Hochschild’s (1983) emotional labour theory, which shows how emotional dissonance, the gap between felt and displayed emotions, can lead to burnout and disengagement.

## Discussion

This study provides critical insights into the gendered dynamics of outsourced customer service work in Romania, revealing how organisational structures and cultural norms intersect to shape workplace inequalities. The findings underscore three interrelated dimensions: gendered occupational segregation, emotional labour expectations, and structural mechanisms of inequality.

First, the gendered distribution of roles reflects the entrenched patterns of vertical segregation. Women constitute most entry-level CSR positions (72%), while men dominate managerial roles (73%). This imbalance aligns with Acker’s (1990), which posits that organisational structures reproduce gender hierarchies through role allocation and promotion practices. Despite women’s numerical dominance, their limited representation in leadership positions illustrates the persistence of systemic barriers to career progression (Scholarios and Taylor, 2011). This echoes Ortiz’s (2022) study, which highlights the historical feminisation of customer service roles and the expectation that women perform invisible emotional labour.

Second, the findings of the ethnographic study clearly demonstrate the disproportionate emotional burden on CSR women. Emotional labour, defined as the regulation of feelings and expressions to meet organisational display rules (Hochschild, 1983), is not only central to customer service work but also profoundly gendered in its expectations and recognitions. Female CSRs are expected to exhibit greater emotional resilience, adaptability, and self-sacrifice than their male counterparts are. For example, Iulia’s narrative illustrates this expectation, and her quote reflects how women internalise organisational norms that prioritise emotional availability and flexibility, often at personal cost. Such expectations align with gendered socialisation processes that position women as natural caregivers and communicators (Carter, 2014). In contrast, male CSRs frame their experiences around skill acquisition and strategic growth, as seen in Samuel’s and Lucian’s accounts. These contrasting narratives reflect symbolic interactionist insights, wherein gendered meanings shape workplace expectations and recognition: women’s emotional contributions remain undervalued and invisible, whereas men’s technical competence is celebrated (Tang and Xu, 2023). Even when women excel, their achievements are often misinterpreted. High-performing female CSRs, such as Iulia, are sometimes perceived as arrogant rather than exemplary, reflecting a double bind in which women must excel but remain modest to avoid social penalties. Meanwhile, men’s assertiveness and ambition are viewed positively, reinforcing gendered stereotypes of leadership. Women are disproportionately expected to suppress negative emotions and maintain their composure under duress, even when facing verbal abuse. This institutional expectation to absorb abuse without retaliation exemplifies the feminisation of affective labour (Zapf et al., 2021).

Additionally, cultural adaptation practices, such as abbreviating names or adopting American accents, impose identity strain, which is disproportionately normalised for women. When such “soft skills” are treated as natural attributes rather than learned competencies, they become easier to standardise, monitor, and extract as organisational value. These findings confirm that emotional labour is not a neutral organisational requirement but a gendered mechanism of control and value extraction. Women’s emotional adaptability is treated as an implicit job requirement, whereas men’s contributions are framed as being strategic and technical. This dynamic perpetuates structural inequalities, reinforcing occupational segregation and limiting women’s access to recognition and advancement in customer support.

Third, ethnographic evidence shows that organisational mechanisms in Romanian call centres exacerbate inequality through deskilling, credential inflation, and reliance on social capital. Organisational mechanisms exacerbate inequality through de-skilling, credential inflation and reliance on social capital. CSR roles are highly standardised and technologically controlled, reducing autonomy and creativity. Tasks are scripted and mediated through software systems, transforming CSRs into task executors rather than problem solvers. This routinisation leads to the underutilisation of graduate skills, creating frustration and diminishing job satisfaction among CSRs employees. These mechanisms intersect with gendered expectations, reinforcing women’s structural disadvantages. Women, who dominate CSR roles, experience deskilling disproportionately because their educational capital has been undervalued. This aligns with Taylorist principles (Woodcock, 2017) and supports Acker’s (1990) argument that organisational logic reproduces gender hierarchies through rigid structures.

This study revealed credential inflation; despite requiring minimal technical skills, employers insisted on university degrees, creating education-job mismatches and underutilising graduate capital. The literature confirms this trend, arguing that credential inflation undermines career progression and fosters precarious employment (Tomlinson, 2023). Recruitment practices privileging employee referrals reinforce closed networks, often male-dominated, thereby excluding women and marginalised groups from the workforce. This mechanism reflects Bourdieu’s (1986) concept of social capital and Rubineau and Fernandez’s (2015) findings on gendered referral networks. Induction processes further perpetuate gender bias, as unpaid shadowing and mentoring, tasks disproportionately performed by women, remain unrecognised, whereas men exploit these periods for disengagement (Guy and Azhar, 2018).

Ethnographic evidence shows that emotional dissonance (the gap between felt and displayed emotions) and identity strain (the pressure to alter or suppress aspects of one’s identity) intensify the precariousness and stress experienced by CSRs, particularly women. CSRs routinely suppress genuine emotions to meet organisational display rules, even under verbal abuse, as illustrated by Lucian and Ion’s accounts. Cultural adaptation practices, such as name abbreviation and American accent adoption, impose additional identity burdens, disproportionately affecting women (Humphrey et al., 2015). These findings resonate with Hochschild’s (1983) emotional labour theory, highlighting how affective performance is commodified yet uncompensated. Both emotional dissonance and identity strain amplify existing structural challenges. They compound deskilling, as emotional regulation becomes a hidden requirement rather than a recognised skill. They intensify credential inflation, as emotional adaptability is demanded in addition to academic qualifications. They intersect with social capital reliance, as women lacking strong networks face additional pressure to conform to cultural and emotional norms to retain their positions. Feminist theory critiques these dynamics as systemic undervaluation of women’s emotional and cultural labour, framing them as “natural” rather than skilled work. Symbolic Interactionism explains how micro-level interactions (e.g., customer expectations, team leader advice) reinforce gendered norms of emotional control and identity adaptation.

These organisational mechanisms collectively reinforce gendered precarity: deskilling strips work of complexity, reducing opportunities for skill recognition; credential inflation traps women in low-status roles despite high educational attainment; and social capital reliance privileges male networks, limiting women’s mobility. Gendered Organisation Theory (Acker, 2012) illuminates how organisational logic embeds gender in everyday practices, from recruitment to task allocation.

## Conclusion

The outsourced service sector in Romania, particularly in call centres, systematically perpetuates gender inequality and precarious employment despite its contribution to economic growth. Our conclusions are based on several key findings. There is a stark vertical segregation, with women overwhelmingly concentrated in low-status, entry-level CSR roles (72%) and men dominating managerial positions (73%). This structural imbalance limits CSR women’s career advancement opportunities. This signifies that, despite high female participation, structural barriers (a glass ceiling) prevent women’s upward mobility, reinforcing traditional gender hierarchies within modern corporate structures. This study reveals that emotional labour is a highly gendered expectation. Female CSRs are required to demonstrate greater emotional resilience, adaptability, and self-sacrifice, whereas men’s contributions are framed around technical skills and strategic growth. This invisible and uncompensated work is often taken for granted, devaluing women’s contributions and reinforcing stereotypes that limit their career progression.

Organisational practices such as deskilling (through task standardisation) and credential inflation (requiring degrees for low-skill jobs) lead to the underutilisation of the educated workforce. This creates job dissatisfaction and burnout, particularly affecting young, educated women who predominate CSR roles. This implies a significant waste of human capital, particularly affecting educated young women who are channeled into these roles. The requirement of a university degree for jobs that do not need one traps educated individuals in low-mobility and precarious employment. This has broader economic implications, suggesting that educational attainment does not guarantee meaningful employment, especially in the outsourced services sector.

Recruitment through closed social networks, uncompensated mentoring by female CSR employees, and high-pressure work environments that demand emotional suppression contribute to an ecosystem where gender inequities are not only present but are actively reproduced within CSR roles. Informal, unpaid mentoring and shadowing are disproportionately performed by women, representing invisible and unacknowledged labour. This not only exploits female employees but also limits their opportunities for advancement, as their developmental work goes unrecognised. The rigid, high-pressure work environment, combined with the expectation to suppress emotions (emotional dissonance) and alter one’s identity (identity strain), creates significant psychological stress. This implies that such work structures are unsustainable and detrimental to employee wellbeing, contributing to high attrition rates and mental health issues.

This ethnographic study primarily concentrated on a single company, Global-Corp, while incorporating supplementary observations from other call centres in Romania. Although this approach provides rich contextual insights into workplace dynamics, its findings may not be entirely generalisable to the broader Romanian Business Services Sector and MNCs. The organisational culture, management styles, and operational practices at Global-Corp differ significantly from those of other MNCs, limiting their applicability. Additionally, this study places gender as the central axis of inequality, acknowledging its profound impact on the workplace experience. However, by focusing solely on gender, we recognise the need for future research to adopt an intersectional perspective. Other identity dimensions, such as age, disability, race, and socioeconomic status, may compound or reshape the experiences of precariousness and emotional labour. Investigating these intersections could provide a more nuanced understanding of vulnerability and inequality in outsourced call centre services.

This study finds that call centres operate as modern workplaces that, through their structure and daily operations, perpetuate traditional gender hierarchies, create insecure career paths, and impose significant psychological burdens on employees, particularly women. The arguments presented in our study have profound implications for understanding contemporary labour markets, especially within the outsourced service sector in emerging economies such as Romania. The analysis reveals how organisational practices and societal norms intersect to sustain gender inequality and precarious working conditions for educated young people, especially women. The primary argument of this study is that call centres are not gender-neutral workplaces; rather, they are environments where gender inequality is actively produced and reinforced.

## Data Availability

The datasets presented in this study can be found in online repositories. The names of the repository/repositories and accession number(s) can be found at: https://doctorat.ubbcluj.ro/ro/sustinerile-publice-ale-tezelor-de-doctorat/?an=2024&luna=0&facultate=0&domeniu=0.
